# The evolutionary and transmission dynamics of HIV-1 CRF08_BC

**DOI:** 10.1371/journal.pone.0310027

**Published:** 2024-09-06

**Authors:** Xingguang Li, Nídia S. Trovão

**Affiliations:** 1 Ningbo No.2 Hospital, Ningbo, China; 2 Guoke Ningbo Life Science and Health Industry Research Institute, Ningbo, China; 3 National Institutes of Health, Fogarty International Center, Bethesda, Maryland, United States of America; Centers for Disease Control and Prevention, UNITED STATES OF AMERICA

## Abstract

HIV-1 CRF08_BC is a significant subtype in China, though its origin and spread remain incompletely understood. Previous studies using partial genomic data have provided insights but lack comprehensive analysis. Here, we investigate the early evolutionary and spatiotemporal dynamics of HIV-1 CRF08_BC in China and Myanmar using near-complete genome sequences. We analyzed 28 near-complete HIV-1 CRF08_BC genomes from China and Myanmar (1997–2013). Phylogenetic, molecular clock, and Bayesian discrete trait analyses were performed to infer the virus’s origin, spread, and associated risk groups. Based on Bayesian time-scaled inference with the best-fitting combination of models determined by marginal likelihood estimation (MLE), we inferred the time to the most recent common ancestor (TMRCA) and evolutionary rate of HIV-1 CRF08_BC to be at 3 October 1991 (95% HPD: 22 February1989–27 November 1993) and 2.30 × 10^−3^ substitutions per site per year (95% HPD: 1.96 × 10^−3^–2.63 × 10^−3^), respectively. Our analysis suggests that HIV-1 CRF08_BC originated in Yunnan Province, China, among injecting drug users, and subsequently spread to other regions. This study provides valuable insights into the early dynamics of HIV-1 CRF08_BC through combined genomic and epidemiological data, which may inform effective prevention and mitigation efforts. However, the limited genomic data influenced the extent of our findings, and challenges in collecting accurate risk group information during surveillance were evident.

## Introduction

Pathogen genomic data can be used in phylogenetic tree reconstruction to determine genealogical relationships between sampled viruses, and provides an additional layer of resolution to determine relationships between cases, beyond that of traditional epidemiology [1–4]. Viral sequence data and respective metadata in association with molecular epidemiological techniques can be used to infer the evolutionary rate and the time of the most recent common ancestor (TMRCA) of pathogens for which little is known about their dynamics, as demonstrated for HIV-1 circulating recombinant form (CRF) 01_AE [5]. Such data also allow the inference of clusters of closely related viruses within a particular surveillance area of interest [6] or among different populations [7], aspects that can be challenging through traditional epidemiology. Viral genetic data can be used to distinguish viral introduction events from endemic transmission. If transmission chains have already been established in a particular surveillance area, then public health policies that seek to reduce the pathogen transmission from other areas are likely to be less effective in reducing case counts. For instance, many travel bans and border restrictions targeting South Africa, that raised the alarm on 24 November 2021 about the SARS-CoV-2 Omicron variant of concern (VOC), were possibly not as effective due to many countries having already detected Omicron circulating in *loco* [2]. Viral genetic data can also be used to develop methods to answer questions that were challenging before. For instance, the efficient and scalable computational phylogenetic inference methods that were developed in response to SARS-CoV-2 [8,9], and the incorporation of travel history in discrete phylogeographic models has shown that some SARS-CoV-2 lineages had already spread to unsampled regions, which in turn can improve the accuracy of phylogenetic inference [10–12]. However, ignoring recombination can lead to biased and inaccurate phylogenetic and phylodynamic inferences [13–15].

HIV-1 CRF08_BC is one of the five main HIV-1 subtypes and circulating recombinant forms in China, along with HIV-1 CRF07_BC, CRF01_AE, CRF55_01B, and B’ (Thai B) [16]. A previous study showed that HIV-1 CRF08_BC was the fourth prevalent genotype in China, accounting for 6.60%, and the first, second, and third prevalent genotypes in China were HIV-1 CRF01_AE (39.69%), CRF07_BC (20.47%), and subtype B (17.50%), respectively [17]. HIV-1 CRF08_BC is the second CRF that was discovered in China, and it is thought to have emerged through the recombination of HIV-1 subtypes B’ and C among injecting drug users (IDU) [18–20]. Many studies have explored the origin, spread, evolutionary history, and factors driving the dispersal of HIV-1 CRF08_BC [16,21–23]. A previous study estimated the mean TMRCA of HIV-1 CRF08_BC ranging from 1989.3 to 1990.3, and the mean evolutionary rate ranged from 1.7 × 10^−3^ to 1.8 × 10^−3^ substitutions per site per year using partial *gag-pol* genes under different evolutionary models [23]. Feng Y et al. estimated the TMRCA of HIV-1 CRF08_BC at around 1992, with a mean evolutionary rate ranging from 2.23 × 10^−3^ to 6.36 × 10^−3^ substitutions per site per year using partial *gag-pol-env* genes [22]. In Liu et al. [24], the authors performed a phylogeographic analysis of the gag-pol genes, which identified Yunnan as the possible origin of CRF08_BC. However, all of the aforementioned studies use partial or sub-genomic regions, which ignores patterns that are imprinted in other longer genomic regions. Furthermore, the studies lack a comprehensive exploration of best performing models to achieve an accurate phylodynamic reconstruction of CFR08_BC [25].

In the present study, we aim at filling this important gap in our understanding of the dynamics and timing of HIV-1 CRF08_BC by employing state-of-the-art approaches to investigate the early genomic epidemiology of HIV-1 CRF08_BC based on all available genomes of HIV-1 CRF08_BC sampled from China and Myanmar with sampling dates between 1997–2013 among various risk groups. Our study provides insights into the spatiotemporal dynamics of HIV-1 CRF08_BC in China and elsewhere.

## Materials and methods

### Collation of near-complete genome dataset of HIV-1 CRF08_BC

All available near-complete genomes (HXB2 genome position 1–9719, with minimum fragment length of 8000 bp) of HIV-1 CRF08_BC with known sampling dates and geographic information were retrieved from the Los Alamos National Laboratory (LANL) HIV Sequence Database (https://www.hiv.lanl.gov/content/sequence/HIV/mainpage.html) as of 26 September 2021. ‘Include problematic sequences’ and ‘One sequence/patient’ were unselected and selected, respectively, before download. The final dataset (‘full28’) included 28 publicly available near-complete genomes (sequence length ranging between 8103 bp and 8782 bp) of HIV-1 CRF08_BC sampled from China (Gansu Province, *n* = 1; Guangdong Province, *n* = 2; Guangxi Province, *n* = 4; and Yunnan Province, *n* = 19), and Myanmar (*n* = 2), with known sampling time (1997–2013), and risk groups (heterosexual, Hetero, *n* = 4; injecting drug users, IDU, *n* = 19; mother-to-baby, MB, *n* = 1; sexual undescribed, SU, *n* = 3; and not recorded, NR, *n* = 1). The ‘full28’ dataset of collected sequences was aligned using MAFFT v7.222 [26] and subsequently manually edited using BioEdit v7.2.5 [27]. Multiple sequence alignments were screened for recombination using RDP v4.101 [28,29] and no recombinant sequences were identified.

### Nucleotide substitution model and maximum-likelihood phylogenetic analyses

The best-fit nucleotide substitution model for ‘full28’ was identified according to the Akaike Information Criterion (AIC), Corrected Akaike Information Criterion (AICc), Bayesian Information Criterion (BIC), and Decision Theory Performance-based Selection (DT) with three substitution schemes (24 candidate models) in jModelTest v2.1.10 [30]. With equal/unequal base frequencies (+F), with/without a proportion of invariable sites (+I), with/without rate variation among sites (+Γ) (nCat = 4; Γ_4_) [31]. ‘Maximum-likelihood (ML) tree’ for base tree for the likelihood calculations and ‘BEST’ tree topology search operation models were also selected. ML phylogenetic reconstruction for ‘full28’ was performed using PhyML v3.1 [32] under a general time-reversible substitution model (GTR) with among-site variation (+Γ_4_) and a proportion of invariable sites (+I), designated as GTR+Γ_4_+I, which was selected as the best-fit model for ML inference by the four model selection methods (AIC, AICc, BIC, and DT) using jModelTest v2.1.10 [30]. Node support was estimated using 1 000 bootstrap replicates [33]. The phylogenetic tree and map were visualized and annotated using Microreact [34].

### Temporal signal and time-scaled phylogenetic analyses

Temporal signal analysis of ‘full28’ were performed using TempEst v1.5.3 [35]. The input tree was the ML tree generated using PhyML v3.1 [32] as mentioned above. ‘Best-fitting root’ option was selected. Time-scaled phylogenetic reconstruction of ‘full28’ was performed through a Markov chain Monte Carlo (MCMC) [36] framework implemented in BEAST (Bayesian Evolutionary Analysis by Sampling Trees) v1.10.4 [37], employing the BEAGLE v4.0.0 [38] high-performance computational library to improve performance. In order to explore the best combination models for ‘full28’, we selected seven coalescent tree priors for ‘full28’: constant size [39], exponential growth [40], logistic growth [40], expansion growth [40], Bayesian Skyline [41], GMRF Bayesian Skyride [42], and Bayesian Skygrid [43]. In addition, we explored two clock models: a strict clock and an uncorrelated relaxed clock with log-normal distribution (UCLN) [44], in combination with each tree prior. In each model combination, the molecular clock rate was set with an uninformative continuous-time Markov chain (CTMC) reference prior [45]. Each Bayesian inference was run for 500 million MCMC states, and sampled every 50 000th MCMC states, in order to reach effective sample sizes (ESSs) for all relevant parameters of at least 200, as determined by Tracer v1.7.2 [46]. We performed Bayesian model selection through marginal likelihood estimation (MLE) to determine the combination of molecular clock and coalescent models that best fits the ‘full28’ dataset. To this end, we employed path-sampling (PS) and stepping-stone sampling (SS) [25,47,48] by running 100 path steps each comprising 10 million states, sampling every 1 000th states, with power posteriors determined from evenly spaced quantiles of a beta (0.3, 1.0) distribution [49]. Each model combination was run 3 times to confirm the consistency of each model combination, to a total of 42 independent Markov chains being set up. We extracted the estimates of evolutionary rate and TMRCA for each model combination using Tracer v1.7.2 [46].

### Bayesian discrete trait reconstruction of sampling location and risk group

To identify the transmission patterns and risk group dynamics of HIV-1 CRF08_BC, we used a Bayesian discrete trait analyses (DTA) for two trait types (sampling location, and risk group), as implemented in BEAST v1.10.4 [37]. For sampling location trait, there were 5 states: Gansu, Guangdong, Guangxi, Myanmar, and Yunnan. For the risk group trait, given that the sexual undescribed (SU) risk group being a code for either Hetero or ‘men who have sex with men’ (MSM), we parameterized SU as an ambiguity code for either Hetero or MSM. Therefore, for the risk group trait, there were two schemes of 4 states: Hetero, IDU, MB, and SU for ‘scheme1’, and Hetero, IDU, MB, and MSM for ‘scheme2’. We used the posterior distribution of trees generated from the best model combination determined by PS and SS comparison after discarding the first 10% as burn-in using LogCombiner v1.10.4 [46], as empirical trees. The reconstruction of ancestral states at internal nodes was performed using an asymmetric substitution model [50]. We performed Bayesian stochastic search variable selection (BSSVS) to simultaneously determine which migration rates are zero depending on the evidence in the data and to efficiently infer ancestral states, in addition to providing a Bayes factor support to identify significant non-zero migration rates [50]. The expected number of DTA transitions (known as Markov jumps) between states was estimated using a robust counting approach [51]. Each log file generated by BEAST v1.10.4 [37] was inspected to confirm that ESSs for all relevant parameters were at least 200 using Tracer v1.7.2 [46]. We used TreeAnnotator v1.10.4 to summarize maximum clade credibility (MCC) trees after discarding the first 10% as burn-in [46]. The MCC trees were visualized and annotated using FigTree v1.4.4 (http://tree.bio.ed.ac.uk/software/figtree/). Very strong supported (Bayes factor >150) transition events were visualized using flumap.blue (https://flowmap.blue).

## Results

### Demographic characteristics and clock-like signal analysis of HIV-1 CRF08_BC

The samples of ‘full28’ were primarily from Yunnan Province (19/28, 67.86%) and from the IDU risk group (19/28, 67.86%), as shown in S1 Fig. For ‘full28’, GTR+Γ_4_+I was selected as the best-fit model under the three substitution schemes (24 candidate models) in jModelTest v2.1.10 [30] according to the four model selection methods (AIC, AICc, BIC, and DT), and was used in subsequent phylogenetic analyses.

### Clock-like signal, ML, and time-scaled phylogenetic analyses of HIV-1 CRF08_BC

**Fig 1:**
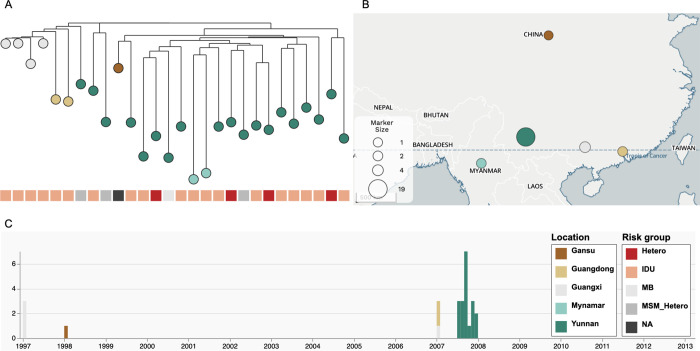
Evolutionary and spatio-temporal history of HIV-1 CRF08_BC. Maximum-likelihood phylogenetic tree of HIV-1 CRF08_BC for ‘full28’. Tip colors indicate sampling locations and heatmap colors indicate different risk groups (A). Map depicts number of sequences collected per location (B). Timeline depicting number of sequences collected per location over time (C). Figure developed and adapted from Microreact^34^. The maps shown in the figure are public domain maps from https://www.openstreetmap.org/about.

ML phylogenetic tree of ‘full28’ showed that all samples from Guangxi Province collected from IDU formed a distinct monophyletic cluster (bootstrap value = 94.1%). The reconstruction also showed that all samples from Myanmar collected from IDU formed a monophyletic cluster with lower bootstrap support (bootstrap value = 40.4%) (Fig 1). We estimated that ‘full28’ had a relatively strong positive temporal signal (*R*^*2*^ = 0.35; correlation coefficient = 0.59) based on linear regression analysis using TempEst v1.5.3 [35] (S2 Fig), even with the limited number of genomes available (S1 Fig). Based on the root-to-tip analysis assuming a strict molecular clock, we estimated the evolutionary rate for the near-complete genome of HIV-1 CRF08_BC to be 4.61 × 10^−3^ substitutions per site per year and the TMRCA of HIV-1 CRF08_BC to be at 10 March 1989.

Based on Bayesian time-scaled phylogenetic reconstruction, the estimated TMRCA dates and evolutionary rates for all parametrizations of the evolutionary dynamics of HIV-1 CRF08_BC for ‘full28’ ranged from 31 May 1982 to 5 October 1991 (95% highest posterior density (HPD) interval: 3 May 1972–27 November 1993) and from 1.72 × 10^−3^ to 2.31 × 10^−3^ substitutions per site per year (95% HPD interval: 1.32 × 10^−3^–2.73 × 10^−3^), respectively (S1 Table). We found that a combination of a non-parametric Bayesian Skygrid coalescent model and an uncorrelated lognormal relaxed (UCLN) molecular clock model was the best-fit model combination for ‘full28’, after comparison among the two clock models and seven coalescent models. Thus, the appropriate TMRCA date and evolutionary rate estimates of HIV-1 CRF08_BC for ‘full28’ with the best-fitting model combination are 3 October 1991 (95% HPD interval: 22 February1989–27 November 1993) and 2.30 × 10^−3^ substitutions per site per year (95% HPD interval: 1.96 × 10^−3^–2.63 × 10^−3^), respectively (S1 Table). Notably, the estimated TMRCA date of HIV-1 CRF08_BC (3 October 1991) was consistent with the result (10 March 1989) based on linear regression analysis using TempEst v1.5.3 [35], and with estimates from previous studies using partial *gag-pol-env* and *gag-pol* genes [22,23]. On the other hand, the Bayesian estimated evolutionary rate of HIV-1 CRF08_BC (2.30 × 10^−3^ substitutions per site per year) was approximately half of that (4.61 × 10^−3^ substitutions per site per year) obtained based on linear regression analysis using TempEst v1.5.3 [35], which assumes a strict molecular clock (S1 Table). Our Bayesian estimates were also generally not consistent with estimates from a previous study (the mean evolutionary rate ranged from 1.7 × 10^−3^ to 1.8 × 10^−3^ substitutions per site per year; the total 95% HPD intervals ranged from 1.3 × 10^−3^ to 2.3 × 10^−3^) using *gag-pol* sequence data [23], since the 95% HPD intervals in both studies mostly not overlap. We also observed that six of the model combinations results were not consistent with the model rank evaluated by PS and SS methods, and that most of them occurred with UCLN molecular clock model parametrizations (S1 Table).

### Ancestral trait estimates of sampling location and risk group of HIV-1 CRF08_BC

**Fig 2:**
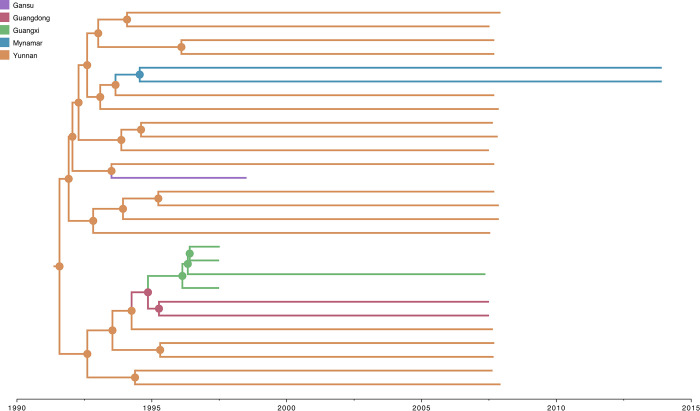
Estimated maximum clade credibility tree of HIV-1 CRF08_BC spatial transmission. Nodes are color-coded by the most probable geographic location of the descendent branches. Color-coded geographic locations are shown on the top left.

**Fig 3:**
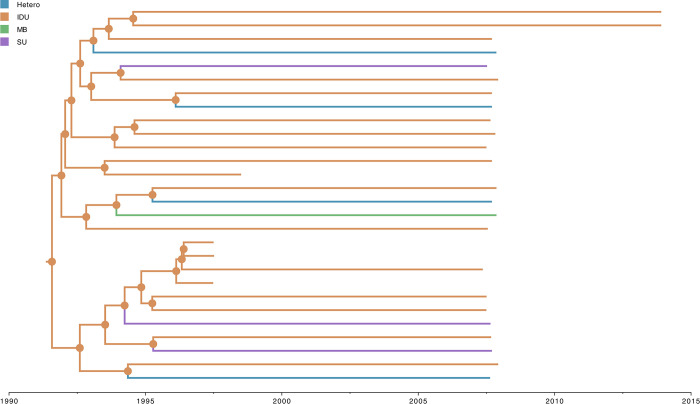
Estimated maximum clade credibility tree of HIV-1 CRF08_BC risk group dynamics. Nodes are color-coded by the most probable risk group of the descendent branches. Color-coded risk groups are shown on the top left.

The selected Bayesian asymmetric DTA of sampling location and risk group for ‘full28’ revealed that the most probable root location of HIV-1 CRF08_BC was in Yunnan Province among IDU populations (posterior probability = 1.0 for both traits) (Figs 2 and 3). The virus appears to spread in a source-to-sink manner, and based on the BSSVS approach, we identified two very strong supported (Bayes factor >150) transition events from Yunnan Province to Gansu Province (median number of Markov jumps: 1; 95% HPD: [0–1]), and from Yunnan Province to Myanmar (median number of Markov jumps: 2; 95% HPD: [0–2] (S3 Fig). We also identified two very strong supported (Bayes factor >150) transition events from IDU to Hetero (median number of Markov jumps: 4; 95% HPD: [3–5]), and from IDU to SU (median number of Markov jumps: 3; 95% HPD: [2–4]) for ‘scheme1’, which is consistent with our findings for ‘scheme2’ where we estimated one very strong supported (Bayes factor >150) transition events from IDU to Hetero (median number of Markov jumps: 4; 95% HPD: [4–6]) (S4 Fig). Our results also revealed that the sample from Gansu Province with NR risk group clustered with IDU with a posterior probability of 0.94. Similarly, all samples from Yunnan Province with SU risk group clustered with IDU with posterior probabilities ranging between 0.84 and 0.85.

## Discussion

**Fig 4:**
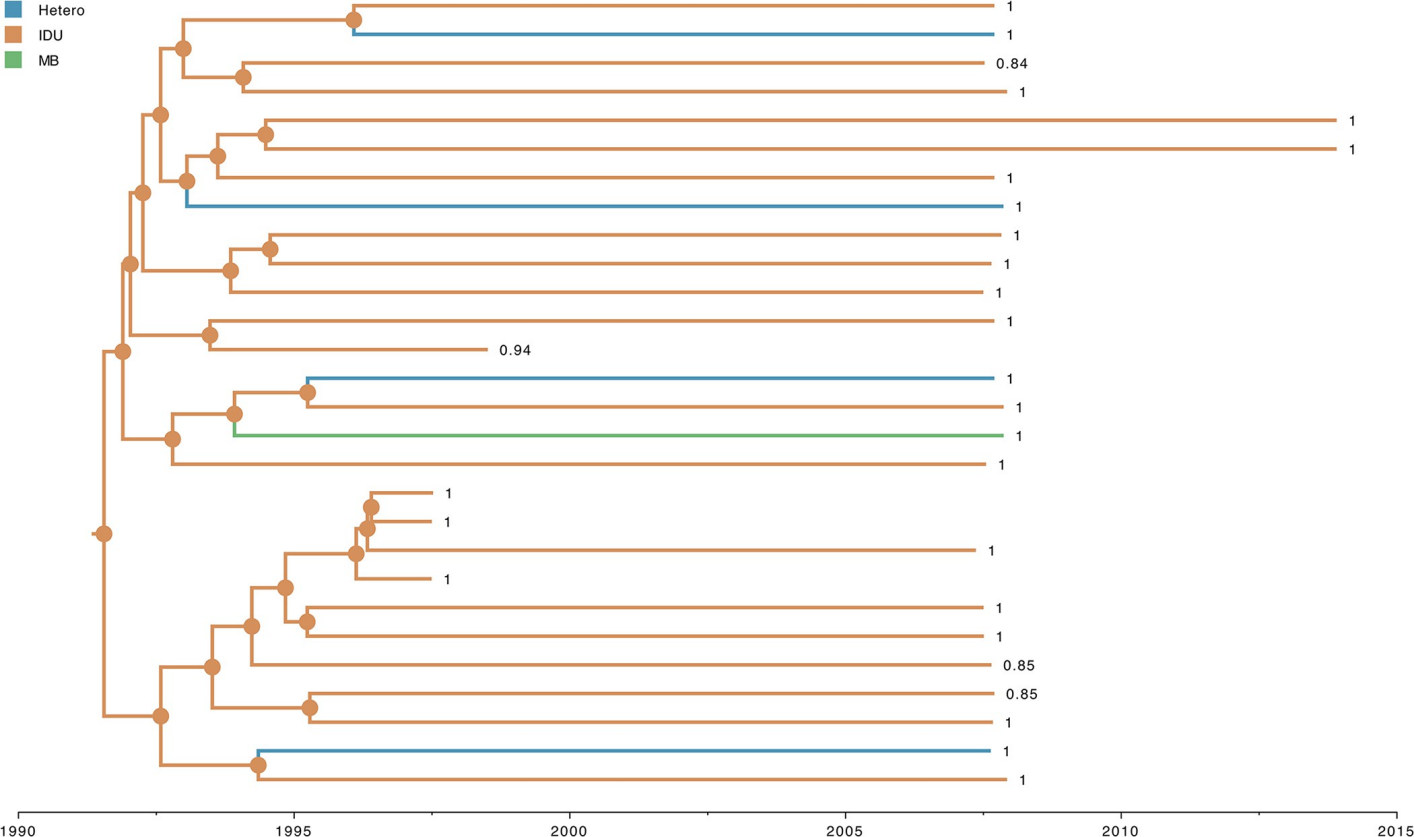
Estimated maximum clade credibility tree of HIV-1 CRF08_BC for NR and SU risk groups. Nodes are color-coded by the most probable risk group of the descendent branches. The highest estimated posterior probability of risk group for each sequence with NR and SU. Color-coded risk groups are shown on the top left.

To investigate the early evolutionary and spatiotemporal history of HIV-1 CRF08_BC, we performed comprehensive evolutionary analyses of 28 near-complete genomes (‘full28’) with sampling location and risk group annotations. The study revealed that HIV-1 CRF08_BC likely originated in Yunnan Province among IDU, in accordance with previous studies [21–23] (Figs 1–4). We estimated that ‘full28’ had a relatively strong positive temporal signal based on linear regression analysis using TempEst v1.5.3 [35] (S2 Fig), even with the limited number of genomes available (S1 Fig). Bayesian analysis of ‘full28’ using an UCLN molecular clock as well as a non-parametric Bayesian Skygrid coalescent model suggested that the estimated TMRCA date of HIV-1 CRF08_BC (3 October 1991) was consistent with the result (10 March 1989) based on linear regression analysis using TempEst v1.5.3 [35], and with estimates from previous studies [22,23]. However, the Bayesian estimated evolutionary rate of HIV-1 CRF08_BC (2.30 × 10^−3^ substitutions per site per year) was approximately half of that (4.61 × 10^−3^ substitutions per site per year) obtained based on linear regression analysis using TempEst v1.5.3 [35], which assumes a strict molecular clock (S1 Table), and was generally consistent with estimates from a previous study (1.9 × 10^−3^ substitutions per site per year; 95% HPD interval: 1.96 × 10^−3^–2.63 × 10^−3^) using *gag-pol* sequence data (strain HXB2; nucleotide 1918 to 2852; 921 bp in length) under an UCLN molecular clock model [23], since the 95% HPD intervals in both studies mostly overlap. We observed that the 95% HPD intervals of TMRCA date and evolutionary rate estimates of HIV-1 CRF08_BC for ‘full28’ were still relatively wide and sensitive to molecular clock and coalescent models (S1 Table), demonstrating that phylodynamic inferences are quite sensitive to priors and models. This is most likely due to the currently limited genomic data available for the early HIV-1 CRF08_BC outbreak period, which hampers accurate inferences for this period. As more patients with HIV-1 CRF08_BC are sampled and more HIV-1 CRF08_BC genomes become available, the additional genomes should make these estimates more robust relative to the choice of molecular clock and coalescent tree prior, and the TMRCA date and evolutionary rate estimates and respective HPD intervals for HIV-1 CRF08_BC will become narrower. The lack of publicly available near-complete genomes of HIV-1 CRF08_BC from LANL HIV Sequence Database (https://www.hiv.lanl.gov/content/sequence/HIV/mainpage.html) sampled since 2013 means that for approximately nine years no new genomes of HIV-1 CRF08_BC have been submitted to LANL HIV Sequence Database (https://www.hiv.lanl.gov/content/sequence/HIV/mainpage.html). This might due to a subdued HIV-1 CRF08_BC epidemic and/or caused by limited molecular surveillance and sequencing.

There are many possible reasons behind this phenomenon, that have also contributed to the limitations in this study. Firstly, HIV-1 CRF08_BC is not recognized as the most prevalent subtype/CRF in China based on the number of infected patients, a spot taken by HIV-1 CRF07_BC [52]; secondly, HIV-1 CRF08_BC is more geographically restricted when compared to HIV-1 CRF07_BC [53]; thirdly, it is challenging and expensive to obtain near-complete genomes of HIV using either Sanger sequencing and next-generation sequencing (NGS) compared to obtain partial or sub-genomic regions of HIV, which could be the reason why not only HIV-1 CRF08_BC, but also other HIV-1 subtypes/CRFs lack publicly available near-complete genomes. Therefore, increased surveillance and technological advances in genomic sequencing of HIV are needed for comprehensive phylodynamic studies that may inform public health interventions.

It is important to note that the genome of HIV-1 CRF08_BC sampled from Gansu Province labeled as risk group NR is estimated to be grouped into IDU with high posterior probability (PP >0.9), therefore, we can infer that this genome of HIV-1 CRF08_BC is likely sampled from an IDU. Despite the small sampling size, this may indicate that patients self-reported as NR in the early spread of HIV-1 CRF08_BC could likely belong to high-risk groups (*i*.*e*., IDU). The mislabeled reports might be a consequence of stigma [54] or fear of legal consequences if patients self-report as IDU. This shows that collecting metadata from HIV-1 patients is a big challenge for epidemiologists. The three genomes of HIV-1 CRF08_BC sampled from Yunnan Province labeled as risk group SU were also estimated to cluster with genomes from IDU with lower posterior probability (PP <0.9), therefore, we cannot confidently infer that these genomes were sampled from IDU or other risk groups, due to the limited number of HIV-1 CRF08_BC genomes available as mentioned above.

In conclusion, this study investigated the origins and spread of HIV-1 CRF08_BC in China and Myanmar using near-complete genome sequences. Our findings suggest the virus originated in Yunnan Province, China, among IDUs and subsequently spread to other areas like Gansu Province and Myanmar, consistent with previous studies. While the estimated emergence date aligns with previous research, the analysis suggests a slower evolutionary rate compared to estimates based on partial genomes. The study highlights the importance of comprehensive genomic surveillance and accurate data collection for understanding the evolution and transmission dynamics of HIV-1 CRF08_BC. Challenges in data collection, particularly regarding risk group information, underscore the need for improved methodologies to gather reliable epidemiological data. By addressing these limitations, future research can provide more reliable insights to guide public health interventions for prevention and controlling HIV-1 CRF08_BC transmission.

## Supporting information

S1 Fig Sampling location and risk group distributions of HIV-1 CRF08_BC. (A) Color-coded bars indicate different sampling locations. (B) Color-coded bars indicate different risk groups.
(TIF)

S2 Fig Linear regression plot of root-to-tip genetic divergence against sampling date of HIV-1 CRF08_BC. Gray-colored line indicates linear regression line.
(TIF)

S3 Fig Estimated migration events between sampled locations for HIV-1 CRF08_BC. Very strongly supported (Bayes factor >150) transition events between sampled locations for HIV-1 CRF08_BC was visualized. Increase in thickness represents stronger viral movement signal. The map shown is made available under the Creative Commons CC0 1.0 Universal Public Domain Dedication and can be found at https://commons.wikimedia.org/wiki/File:BlankMap-World-2009.svg.
(TIF)

S4 Fig Estimated migration events between risk groups for HIV-1 CRF08_BC for schemes 1 and 2. The very strongly supported (Bayes factor >150) transition events between risk groups for HIV-1 CRF08_BC was visualized using a Sankey plot. Increase in thickness represents stronger viral movement signal.
(TIF)

S1 Table Bayesian phylogenetic estimates of evolutionary parameters and model comparison for genome sequences of HIV-1 CRF08_BC under different clock models and coalescent tree priors with three independent runs each. (XLSX)
